# Supplementary material for: Efficient information extraction using LLMs and knowledge distillation: A study on HPV health communication
Source: PLOS Digit Health. 2026 Mar 10;5(3):e0001275. doi: 10.1371/journal.pdig.0001275 (PMC12974803; doi:10.1371/journal.pdig.0001275)
Supplement: S1 Text — (DOCX) [file pdig.0001275.s001.docx]

**States with limited data**

The states for which only top-100 data were fetched from their larger set include: Alabama, Arizona, California, Florida, Georgia, Hawaii, Illinois, Indiana, Iowa, Kansas, Kentucky, Louisiana, Maine, Maryland, Massachusetts, Michigan, Minnesota, Mississippi, Missouri, Nebraska, New Jersey, New York, North Dakota, Ohio, Oregon, Rhode Island, South Dakota, Texas, Vermont, Virginia, Washington, West Virginia, Wisconsin and Wyoming.

**Content Coverage Analysis for States Affected by the 100-Page Retrieval Limit**

After filtering PDFs, documents or spreadsheets, only one state retrieved the maximum 100 pages from the Google Search API: North-Dakota. To assess whether this limit meaningfully constrained content covered, we examined the cumulative count of HPV mentions (‘HPV’ or ‘Human Papilloma-virus’) across the pages ranked by Google’s relevance algorithm and present below.

**Table 1. Cumulative HPV mentions in top-k pages for North-Dakota**

| **K** | **Cumulative HPV Mentions** | **% of Total** |
| --- | --- | --- |
| 20 | 118 | 44% |
| 40 | 158 | 59% |
| 60 | 198 | 74% |
| 80 | 238 | 89% |
| 90 | 253 | 95% |
| 95 | 259 | 97% |
| 100 | 266 | 100% |

The data show diminishing returns with additional pages. The top 20 pages (20% of content) capture 44% of all HPV mentions, while the top 60 pages (60% of content) capture 74%. Conversely, the final 40 pages contribute only 26% of total mentions despite representing 40% of retrieved pages. As expected with Google's relevance-based ranking, lower-ranked pages contain fewer substantive references to the query topic. This likely reflects HPV mentions appearing in less relevant contexts (e.g., navigation elements, brief mentions in broader health content, or page metadata) rather than dedicated HPV-focused content. The 100-page limit does not meaningfully constrain coverage of HPV-related content for North Dakota and is unlikely to underestimate coverage for other states that retrieved fewer pages.
