# Supplementary material for: Efficient information extraction using LLMs and knowledge distillation: A study on HPV health communication
Source: PLOS Digit Health. 2026 Mar 10;5(3):e0001275. doi: 10.1371/journal.pdig.0001275 (PMC12974803; doi:10.1371/journal.pdig.0001275)
Supplement: S2 Text — (DOCX) [file pdig.0001275.s002.docx]

**Experimental Paradigm**

Model performance was evaluated using precision (P), recall (R), and F1-score (F1) metrics. Overall performance was assessed as the micro-average F1 across all labels.

For KD experiments, a random sample of 1,000 text instances from the full set of primary and secondary pages (excluding samples from Targeted HPV Dataset) were selected. The teacher model generated corresponding soft labels for these samples that were used to train the student models. The dataset comprises 1,000 samples, of which 320 (32%) contain at least one positive label. The relative sparsity of the training set compared to the test set results from our rigorous curation process of the Targeted HPV dataset, which primarily affected samples from the primary pages used in our validation and test sets. To address this, we supplemented the training data with samples from secondary pages, which typically contained less relevant information, leading to a higher proportion of negative samples. Despite this class imbalance (32:68 ratio), the 320 positive samples were sufficient for effective model learning, particularly for our encoder-only architecture, as demonstrated by the experimental results. Hyperparameters were tuned to maximize the micro-average F1 on the validation set. For the Llama family models, we used a learning rate of 2e-4, batch size of 8, weight decay of 0.01, and trained for 5 epochs. RoBERTa hyperparameters included a learning rate of 2e-5, batch size of 16, weight decay of 0.001, and 30 training epochs.
