# Supplementary material for: Efficient information extraction using LLMs and knowledge distillation: A study on HPV health communication
Source: PLOS Digit Health. 2026 Mar 10;5(3):e0001275. doi: 10.1371/journal.pdig.0001275 (PMC12974803; doi:10.1371/journal.pdig.0001275)
Supplement: S3 Text — (DOCX) [file pdig.0001275.s003.docx]

**Data Examples**

We present some text samples for some labels to better understand data that requires explicit vs implicit reasoning as discussed in Error Analysis.

Prevent Spread: "*Why get the HPV vaccine?*

*HPV types that cause most HPV cancers and genital warts have dropped 88 percent among teen girls and 81 percent among young women*."

Sexual Spread: “*Who should get the HPV vaccine?*

*Vaccination is not recommended for everyone older than age 26 years. However, some adults age 27 through 45 years who are not already vaccinated may decide to get the HPV vaccine after speaking with their healthcare provider about their risk for new HPV infections and the possible benefits of vaccination. HPV vaccination in this age range provides less benefit. Most sexually active adults have already been exposed to HPV, although not necessarily all of the HPV types targeted by vaccination*.”

Cancer Prevention: “*When Should My Child Get the HPV Vaccine?*

*HPV vaccination is recommended at ages 11-12 years. HPV vaccines can be given starting at age nine years. Vaccines protect your child before they are exposed to an infection. That’s why we give HPV vaccination earlier, rather than later, to protect them long before they are exposed. Also, if your child gets the shot now (before they turn 15), they will only need two shots. If you wait until your child is older, they will need three shots. All preteens need HPV vaccination, so they are protected from HPV infections that can cause cancer later in life. Teens and young adults through age 26 years who didn’t start or finish the HPV vaccine series also need HPV vaccination.*”
