# Supplementary material for: Efficient information extraction using LLMs and knowledge distillation: A study on HPV health communication
Source: PLOS Digit Health. 2026 Mar 10;5(3):e0001275. doi: 10.1371/journal.pdig.0001275 (PMC12974803; doi:10.1371/journal.pdig.0001275)
Supplement: S1 Table — (DOCX) [file pdig.0001275.s004.docx]

**Annotation Guidelines**

Table 1. HPV Guidelines with Questions, Labels, and Evaluation Criteria

| **Question** | **Label** | **Guidelines** |
| --- | --- | --- |
| Does the text indicate HPV infection is asymptomatic? | Asymptomatic | Look for explicit or implicit references indicating that HPV infection has no symptoms. |
| Does the text indicate HPV infection cannot be cured? | No Cure | Relevant statements should explicitly indicate that there is no cure for HPV infection |
| Does the text indicate HPV is primarily contracted through sexual contact? | Sexual Spread | Relevant statements should explicitly indicate that HPV is spread mainly through sexual activity. Reference to non-sexual methods of contractions are not applicable. |
| Does the text describe HPV causing cervical cancer? | Cause of Cervical Cancer | Relevant statements should explicitly indicate HPV can cause cervical cancer. References to other cancers are not applicable. |
| Does the text indicate HPV vaccination can prevent cancers stemming from HPV? | Cancer Prevention | Relevant statements should explicitly state that HPV vaccination can prevent cancers caused by HPV. Focus on vaccination as a cancer prevention tool only regardless of what cancer it is. |
| Does the text indicate HPV causing any cancer in general? | Cause of Cancer | Relevant statements should explicitly state that HPV causes any type of cancer apart from Cervical Cancer. Do not consider statements that only mention cervical cancer. The focus should be on HPV causing cancer in general terms. |
| Does the text indicate HPV vaccination can prevent spreading HPV? | Prevent Spread | Relevant statements should explicitly state that HPV vaccination can prevent the spread of the virus. This includes any mention of vaccination preventing the contraction of HPV, or stopping the development of HPV infection as it indirectly prevents its spread. |
| Does the text indicate HPV vaccination as highly effective at preventing HPV and/or related cancers? | Highly Effective | Relevant statements should explicitly state that the HPV vaccine is highly effective in preventing the virus. References with synonymous words of high effectiveness of HPV vaccine should be captured |
| Does the text describe the number of HPV vaccine doses that are needed? | Number Doses | Relevant statements should explicitly address the safety of the vaccine or mention potential side effects from being vaccinated. |
| Does the text describe safety concerns and potential side effects of HPV vaccination? | Side Effects | Relevant statements should explicitly indicate how safe the vaccine is or address the side-effects that could incur from being vaccinated. |
| Does the text recommend HPV vaccination for children? | Rec-Children | Relevant statements should explicitly recommend HPV vaccination for children (9-15 in age). Ensure recommendations for children are clearly identified and not confused with mere suggestions. |
| Does the text recommend HPV vaccination for both males and females? | Rec-Male Female | Relevant statements should explicitly recommend HPV vaccination for both males and females regardless of their age. Look for phrases indicating that vaccination is advised, suggested, or endorsed for both genders. |
| Does the text recommend HPV vaccination? | Stance-Recommend | Relevant statements should explicitly recommend HPV vaccination to any age group/gender instead of suggesting. |
| Does the text describe HPV vaccination as optional? | Stance-Optional | Relevant statements should explicitly describe HPV vaccination as optional or a personal choice. Look for phrases indicating that vaccination is not mandatory, such as "can choose," ,"may get vaccinated","is optional," "personal decision," or similar expressions. |
| Does the text reference reputable sources (e.g. CDC, ACS, AAP)? | Source Credibility | Relevant statements should mention about HPV/ HPV vaccination and explicitly mention reputable sources such as the CDC, ACS, AAP, or other well-known health organizations. |

Table 2. Questions, Labels and their corresponding CoT questions

| **Question** | **Label** | **CoT Questions** |
| --- | --- | --- |
| Does the text indicate HPV infection is asymptomatic? | Asymptomatic | No CoT questions |
| Does the text indicate HPV infection cannot be cured? | No Cure | No CoT questions |
| Does the text indicate HPV is primarily contracted through sexual contact? | Sexual Spread | 1) Does the text mention particular activities such as vaginal intercourse, anal sex, oral sex, or genital-to-genital contact  2) Does the text explicitly assert a causal relationship between HPV infection and activities such as vaginal intercourse, anal sex, oral sex, or genital-to-genital contact?​ |
| Does the text describe HPV causing cervical cancer? | Cause of Cervical Cancer | 1) Does the text mention Cervical Cancer?  2) Does the text assert a causal relationship between HPV and Cervical Cancer? |
| Does the text indicate HPV vaccination can prevent cancers stemming from HPV? | Cancer Prevention | 1) Does the text explicitly state that HPV vaccination can prevent cancers caused by HPV?  2) Does the text focus on HPV vaccination as a cancer prevention tool regardless of what cancer it is? |
| Does the text indicate HPV causing any cancer in general? | Cause of Cancer | 1) Does the text mention any cancer other than Cervical Cancer?  2) Does the text assert a causal relationship between HPV and any cancer other than Cervical Cancer? |
| Does the text indicate HPV vaccination can prevent spreading HPV? | Prevent Spread | 1) Does the text show a preventive relationship between HPV vaccination and HPV infection, HPV disease? |
| Does the text indicate HPV vaccination as highly effective at preventing HPV and/or related cancers? | Highly Effective | No CoT questions |
| Does the text describe the number of HPV vaccine doses that are needed? | Number Doses | No CoT questions |
| Does the text describe safety concerns and potential side effects of HPV vaccination? | Side Effects | No CoT questions |
| Does the text recommend HPV vaccination for children? | Rec-Children | 1) Does the text mention children or people under the age of 18?  2) Does the text assert a recommendation relationship between HPV vaccine and people? |
| Does the text recommend HPV vaccination for both males and females? | Rec-Male Female | 1) Does the text mention both male and female genders?  2) Does the text assert a recommendation relationship between HPV vaccine and people? |
| Does the text recommend HPV vaccination? | Stance-Recommend | No CoT questions |
| Does the text describe HPV vaccination as optional? | Stance-Optional | No CoT questions |
| Does the text reference reputable sources (e.g. CDC, ACS, AAP)? | Source Credibility | No CoT questions |

Table 3. Student Model Architectures, their prompting strategies and corresponding Teacher models

| **Student Model**  **Architecture** | **Teacher Model Prompting Strategy** | **Prompting Strategy/ Output from the model** |
| --- | --- | --- |
| LLM Baseline | Zero-shot + Guidelines | Input:  **System**: You are a helpful assistant  **Question**: <Question>  **Text**: <Text>  **Guideline**: <Relevant Guideline>  **Task**: <Task>  Output: <Yes/ No> |
| LLM + Rationale | Zero-shot + Guidelines +  Rationale | Input:  **System**: You are a helpful assistant  **Question**: <Question>  **Text**: <Text>  **Guideline**: <Relevant Guideline>  **Task**: <Task>  Output: Rationale + <Yes/ No> |
| LLM + CoT | Zero-shot + Guidelines | Input:  **Task**: <Task>  **Example:**  **example specific input text:** <text>  **example specific question:** <question>  **Output:**  **CoT1:** *<CoT1>*  **CoTAnswer1**: *<CoT1 Answer>*  **CoT2:** *<CoT2>*                                  **CoTAnswer2:** *<CoT2 Answer>*  **Final Answer:** *yes*   **Now, here’s your input for the same task. Represent the answer in the exact form presented above:**  **Text**: <Actual Text>  Output: CoT + <Yes/ No> |
| Encoder Baseline | Zero-shot + Guidelines | Input:  **Text**: <Text>  Output: Vector of size 15 |
| Encoder + CoT | Zero-shot + Guidelines | Input:  **Text**: <Text>  Output: Vector of size 26 |
