# Supplementary material for: Efficient information extraction using LLMs and knowledge distillation: A study on HPV health communication
Source: PLOS Digit Health. 2026 Mar 10;5(3):e0001275. doi: 10.1371/journal.pdig.0001275 (PMC12974803; doi:10.1371/journal.pdig.0001275)
Supplement: Attachment — Submitted filename: Response To Reviewers.pdf [file pdig.0001275.s006.pdf]

Response Document

We carefully read the reviewer comments and have edited the manuscript to address as many points as possible. We interleaved our responses with the reviewers' comments below, to provide context for the responses. A version of the manuscript indicating the associated changes is provided with the revised manuscript.

Reviewer 1

1) Data and Model Accessibility: The new annotated dataset is a fantastic resource for the community. To fully align with the open science goals of PLOS Digital Health and ensure other researchers can build on this important work, the authors should consider depositing the dataset and the final trained RoBERTa model in a public repository. The journal's policy requires data to be fully available before publication (please see the Data Availability policy).

Response: We added a link to a GitHub repository with the annotated dataset and all associated code (knowledge distillation, evaluation, collecting data, etc.). The trained model is also released to HuggingFace and the link for downloading the trained model is presented in our GitHub repository.

2) Exploring the Data Collection Limits: The case study analyzing content across state DOH websites is a compelling application of the model. To make the conclusions from the heatmap even more robust, it might be helpful to expand the discussion on the potential impact of the Google Search API's 100-result limit. It's possible that for states with a lot of webpages, this limit might result in an underestimation of their topic coverage. Perhaps a small validation, like a deeper manual search for one or two states that appear to have low coverage, could help quantify this and really strengthen this part of the paper.

Response: This suggestion is both helpful and insightful. After filtering PDFs and attachments, only one state (North Dakota) retrieved the maximum 100 pages from the Google Search API. We added a *Content Coverage Analysis for States Affected by the 100-Page Retrieval Limit* section to the Appendix (Table 1) examining the cumulative distribution of HPV mentions across pages ranked by relevance. The analysis shows the top 20% of pages capture 44% of HPV mentions, while the final 40% of pages contribute only 26% of mentions, demonstrating diminishing returns consistent with Google's relevance ranking. Lower-ranked pages contain HPV mentions in less relevant contexts (navigation elements, brief mentions without health context, or page metadata) rather than substantive HPV-focused content. These results confirm that the 100-page limit does not meaningfully constrain content coverage.

3) Expanding on the Error Analysis: It was interesting that the smaller RoBERTa model actually did better on some of the tasks that required more inference, while the larger teacher model was better at pulling out explicitly stated facts. I think the paper would be even more impactful if the authors could expand the discussion with their thoughts on why this might have happened.

Response: We thank the reviewer for their interest in the observed performance differences. We have expanded the Error Analysis section to address this finding. The teacher model's superior performance on explicit fact extraction (e.g., No Cure, Number Doses) aligns with LLMs' strength in matching clearly stated information. However, its relatively weaker performance on inference-based labels parallels the commonly reported "overthinking" problem in LLMs, where excessive reasoning can harm performance on tasks requiring contextual interpretation. Conversely, the student model maintained or enhanced capabilities for interpreting implicit information (e.g., Cancer Prevention, Sexual Spread), suggesting that knowledge distillation may preserve these inference-based capabilities while reducing explicit fact extraction performance. We provide specific examples and further discussion in the Error Analysis section.

4) Typos and using 'Topic Coverage' instead of information completeness:

Response: We agree with the suggestions and have fixed the typos and changed the term 'information completeness' to 'topic coverage'.

Reviewer 2

1) To ensure the claim that the model performs zero-shot classification, the authors should specify the time window of data collection for the HPV data provided by the Department of Health (DOH). The temporal gap between the model's release date and the dataset collection period is critical to demonstrate that the model had no prior exposure to the collected data during pretraining. If the data were gathered before or near the model's training period, there is no guarantee that portions of the data provided by DOH were not included in the model's original training corpus. Therefore, the dataset collection must have occurred well after the release of the Llama 3.1 70B model to validate its characterization as a truly zero-shot evaluation. I think this paper would have been better if the zero-shot concept had been excluded.

Response: We appreciate the reviewer raising this important consideration. Foundation models are trained on vast portions of the publicly available internet, and it is likely that some DOH webpage content was included in pretraining corpora. This exposure through next-token prediction can affect model behavior, such as reducing perplexity on familiar content.

However, domain exposure during pretraining is distinct from task exposure. In standard machine learning terminology, a task is considered 1-shot or n-shot when the model has been trained on one or n labeled examples of that task, or when such examples are provided in-context during prompting [1]. We neither fine-tuned the LLMs on this task nor provided with in-context examples when evaluated for zero-shot performance. Therefore, our evaluation meets the standard definition of zero-shot.

The relatively modest performance across LLMs, particularly smaller ones, supports that this specific classification task was likely not part of their training objectives. While the models may have encountered HPV content during pretraining, this domain familiarity does not constitute task-specific training. We feel the zero-shot characterization is appropriate and consistent with standard usage in the literature.

[1]. Brown, T. B., Mann, B., Ryder, N., Subbiah, M., Kaplan, J., Dhariwal, P., Neelakantan, A., Shyam, P., Sastry, G., Askell, A., et al. (2020). Language Models are Few-Shot Learners. Advances in Neural Information Processing Systems.

Reviewer 3

1) The discussion should better correlated computational findings with practical implications for health-related communication strategies as well as for vaccine uptake.

Response: We agree with the reviewer that framing health-related communication strategies as having positive outcomes, including increased vaccination uptake, more clearly motivates the purpose of this work. We have incorporated this perspective into the Introduction as part of our motivation and have revisited it in the Discussion section.

2) With regards to the ethical section, it would be appropriate to emphasize risk of bias propagation assessments in downstream systems

Response: We appreciate this thoughtful feedback. We have substantially expanded the Ethical and Legal Considerations section to emphasize bias propagation risks in downstream systems. The updated section addresses: 1) quantitative bias associated with differential model performance across categories, 2) identification of bias sources, including variations in reading level, linguistic complexity, cultural framing, disparities in digital access, and information quality across webpages, and 3) explicit guidance that

deployed systems require these sources of bias to be systematically examined and mitigated.

3) I would call the authors attention to the data availability statement, particularly specifying the exact format and process for the dataset access to increase transparency and reproducibility.

Response: As mentioned in a previous response, we have released the dataset along with the model training and evaluation scripts through our GitHub repository.

4) Some sentences in the results are too lengthy and could be shortened for better clarity.

Response: We thank the reviewer for the suggestion. We have tried our best to shorten certain sentences without losing clarity.

5) Clinically speaking, authors might emphasize the translational rhetoric through the articulation of how improved quality of health communication has been proven to influence measurable epidemiological parameters (including vaccination coverage)

Response: We believe this feedback closely aligns with the reviewer's first comment. To address it, we have cited relevant prior work that highlights this point and have framed it more clearly as part of the motivation for our study.

6) As DOH platforms are utilized by contrasting groups, a short paragraph approaching disparities in digital access, health literacy, and cultural tailoring of online interactions would enrich the discussion

Response: We expanded the Ethical and Legal Considerations section to address these important disparities. Specifically, we now discuss how variations in reading level, linguistic complexity, and cultural framing of health content may affect model predictions in ways that disadvantage certain populations, with appropriate suggestions added. We also emphasize that disparities in digital access vary across populations and that any deployed system should be designed with these inequities explicitly in mind. We note that if deployed operationally, these sources of bias propagation should be systematically examined and mitigated.

7) This is critical - the authors should stress that the proposed framework could be definitely extended to NCD to assess the accuracy and impact of online health communication.

Response: We thank the reviewer for this astute suggestion. We have incorporated this perspective into the *Broader Implications* section, discussing how our work can be extended to other diseases, including non-communicable conditions.

Reviewer 4

1) The paper provides an extensive overview of the data collection and annotation process, but a few methodological clarifications would improve reproducibility: Dataset transparency: The Targeted HPV Dataset (n=400) is mentioned as available upon request, but a public link or structured data statement (e.g., schema of each variable, annotation format) would strengthen the data availability claim.

Response: As discussed in a previous reviewer response, we release the data and model training and evaluation scripts through our GitHub repo.

2) Annotation guidelines: While Appendix Table 1 provides questions and labels, inter-annotator agreement (IAA = 0.87 F1) lacks details on Cohen's κ or whether disagreements were resolved via adjudication. Clarifying this would improve transparency.

Response: We thank the reviewer for this suggestion. In addition to reporting the F1 score for IAA, we have now also included the Cohen's Kappa score at the same location in the paper.

3) Sampling bias: Since the dataset disproportionately represents primary pages, the authors should discuss potential sampling bias and how it might affect generalizability to broader DOH content.

Response: To ensure sufficient positive samples for annotation, we manually identified each state's primary HPV-focused webpage and sampled content from these pages and their directly linked secondary pages. This strategy deliberately prioritized HPV-relevant content over random sampling. We acknowledge this creates sampling bias toward HPV-focused content. However, even with this intentional bias, the Targeted HPV Dataset contains substantial negative samples and demonstrates considerable variation in information density. Many HPV-related pages lack target elements, while others contain multiple elements. While this sampling strategy limits generalizability to non-HPV DOH content, it was necessary to create a balanced dataset with adequate positive examples for each label.

4) The RoBERTa Large model (student) achieving near-parity with Llama 3.1 70B is impressive, yet the manuscript lacks comparison with other compact encoder-based models (e.g., DistilBERT, BioClinicalBERT, or PubMedBERT). A brief ablation or

reference to prior work showing similar benchmarks would contextualize the advantage of the KD approach.

Response: We thank the reviewer for this valuable suggestion. We have added results from DistilBERT to the *Experiments* section to provide comparison with another compact encoder-based model.

We did not conduct experiments with BioClinicalBERT or PubMedBERT because our dataset primarily consists of layperson oriented public health content rather than clinical documentation. These domain specific models are designed to yield substantial improvements when processing medical terminology and clinical narratives, which differ from the more general health communication language found on Department of Health websites. Given this mismatch between the models' pretraining domain and our content, we focused on general purpose encoders such as DistilBERT and RoBERTa.

5) The use of bootstrap significance testing is commendable. However, please specify the confidence intervals (e.g., 95%) and provide effect sizes for key comparisons (Teacher vs Student). The F1 differences are small ($\Delta = 0.03$); providing standard deviation or variance across labels would help readers assess robustness. It would be useful to visualize label-level performance variability using a bar or radar plot to show where the student model outperforms the teacher.

Response: We have added the confidence interval (95%) and have provided the label-level variance for both the teacher and student models.

6) The qualitative error analysis section is insightful but could be expanded. Specifically: Provide 2–3 concrete text examples illustrating why the student model performs better on inferred information (e.g., implicit causal relationships)

Response: We have added concrete text examples to the Appendix to illustrate the quality of content requiring inference. While complete webpage excerpts can be lengthy, we provide representative examples demonstrating implicit tasks where the student model outperforms the teacher model. These examples clarify the nature of inference-based classification in our dataset.

7) Include a short discussion of model interpretability (e.g., use of attention maps, SHAP, or LIME) to demonstrate how the model identifies key phrases or constructs during evaluation.

Response: We appreciate this suggestion and agree that interpretability analysis would provide valuable insights into model decision-making. However, this falls outside the scope of our current work, which focuses on establishing a knowledge distillation pipeline for multi-label health content classification and analyzing performance variation between teacher and student models. Interpretability analysis (e.g., attention visualization, SHAP values) would constitute a substantial additional investigation, as different classification labels likely rely on distinct linguistic patterns and contextual cues that would require systematic analysis across all labels. This as an important direction for future work that would complement our findings on the comparative strengths of explicit versus inference-based classification.

8) While the manuscript appropriately acknowledges ethical and legal constraints, two key areas merit deeper discussion: Bias propagation: Beyond acknowledging potential model bias, a brief quantitative analysis (e.g., topic frequency or linguistic bias by state) would demonstrate proactive bias monitoring.

Response: We thank the reviewer for raising this important concern and agree that such analyses would provide valuable insight. However, conducting these experiments would require additional information, such as detailed linguistic and demographic characteristics across states, which falls beyond the scope of the present study.

We recognize the importance of systematically identifying and mitigating different forms of bias. Accordingly, we expanded the Ethical and Legal Considerations section to more clearly discuss the potential for bias propagation in downstream systems. The revised section addresses: (1) quantitative bias arising from differential model performance across categories; (2) potential sources of bias, including variations in reading level, linguistic complexity, cultural framing, disparities in digital access, and differences in information quality across webpages; and (3) the need for systematic evaluation and mitigation of these factors prior to deployment.

While we acknowledge the possibility of bias propagation within our system, rigorously measuring and monitoring such bias constitutes a distinct line of research. The primary objective of our work is to demonstrate an effective knowledge distillation framework for information assessment, and therefore comprehensive bias quantification falls outside the scope of the current study.

9) Data collection ethics: Some DOH websites restricted automated scraping (HTTP 403). The authors should state whether institutional review or data use compliance measures were taken, as per PLOS Digital Health's transparency guidelines.

Response: We scraped only publicly available data from state websites, and any states that imposed restrictions on scraping were excluded from our corpus. We therefore believe that appropriate compliance measures were followed. In addition, we release only the annotated dataset to support reproducibility and further research within the community rather than distributing the full scraped corpus. We also outline a fair use policy of the annotated data for scientific advancement and clarify that the release of the annotated set does not alter or misrepresent the original content.

10) Minor Comments: Grouped citations, defining the 'teacher' and 'student' models in their first mention, consistency in decimal values of scores and using peer reviewed publications where applicable.

Response: We thank the reviewer for the minor comments. We have revised the manuscript accordingly by adding and correcting citations where appropriate, explicitly specifying the selected teacher and student models in the Knowledge Distillation subsection of the Methods section, and reporting all scores to two decimal places in the updated version.
