# Supplementary material for: Efficient information extraction using LLMs and knowledge distillation: A study on HPV health communication
Source: PLOS Digit Health. 2026 Mar 10;5(3):e0001275. doi: 10.1371/journal.pdig.0001275 (PMC12974803; doi:10.1371/journal.pdig.0001275)
Supplement: Attachment — Submitted filename: PDIG-D-25-00609.docx [file pdig.0001275.s005.docx]

Efficient Information Extraction Using LLMs and Knowledge Distillation: A Study on HPV Health Communication

This manuscript presents a well-structured and technically sound study that leverages large language models (LLMs) and knowledge distillation (KD) to evaluate the quality and completeness of HPV-related information across U.S. state Department of Health (DOH) websites. The authors introduce a two-stage framework — first, developing a teacher–student model distillation approach using the Llama family and RoBERTa architectures, and second, applying the distilled model to large-scale web data. The study contributes to both computational methodology and public health informatics by providing an efficient, scalable approach for automated health communication assessment.

I have the following comments.

**Major Comments**

**1.** The paper provides an extensive overview of the data collection and annotation process, but a few methodological clarifications would improve reproducibility:

- Dataset transparency: The Targeted HPV Dataset (n=400) is mentioned as available upon request, but a public link or structured data statement (e.g., schema of each variable, annotation format) would strengthen the data availability claim.
- Annotation guidelines: While Appendix Table 1 provides questions and labels, inter-annotator agreement (IAA = 0.87 F1) lacks details on Cohen’s κ or whether disagreements were resolved via adjudication. Clarifying this would improve transparency.
- Sampling bias: Since the dataset disproportionately represents primary pages, the authors should discuss potential sampling bias and how it might affect generalizability to broader DOH content.

**2.** The RoBERTa Large model (student) achieving near-parity with Llama 3.1 70B is impressive, yet the manuscript lacks comparison with other compact encoder-based models (e.g., DistilBERT, BioClinicalBERT, or PubMedBERT). A brief ablation or reference to prior work showing similar benchmarks would contextualize the advantage of the KD approach.

3. The use of bootstrap significance testing is commendable. However, please specify the **confidence intervals** (e.g., 95%) and provide **effect sizes** for key comparisons (Teacher vs Student). The F1 differences are small (Δ = 0.03); providing **standard deviation or variance across labels** would help readers assess robustness. It would be useful to visualize label-level performance variability using a **bar or radar plot** to show where the student model outperforms the teacher.

4. The qualitative error analysis section is insightful but could be expanded. Specifically:

- Provide 2–3 concrete text examples illustrating *why* the student model performs better on inferred information (e.g., implicit causal relationships).
- Include a short discussion of model interpretability (e.g., use of attention maps, SHAP, or LIME) to demonstrate how the model identifies key phrases or constructs during evaluation.

5. While the manuscript appropriately acknowledges ethical and legal constraints, two key areas merit deeper discussion:

- **Bias propagation:** Beyond acknowledging potential model bias, a brief quantitative analysis (e.g., topic frequency or linguistic bias by state) would demonstrate proactive bias monitoring.
- **Data collection ethics:** Some DOH websites restricted automated scraping (HTTP 403). The authors should state whether institutional review or data use compliance measures were taken, as per *PLOS Digital Health*’s transparency guidelines.

Minor Comments –

1.*Introduction* could be slightly condensed to improve flow. Several citations (e.g., refs. 11–17) could be grouped to streamline readability.

2. The use of “teacher” and “student” models should be defined clearly in the *Methods* section (first mention).

3. Some references (e.g., arXiv citations [43–47]) could be complemented by peer-reviewed publications where available.

4. Ensure consistency in F1 score reporting (some tables use two decimal places, others one).
